# Effects of Consuming Calcium-Rich Foods on the Incidence of Type 2 Diabetes Mellitus

**DOI:** 10.3390/nu11010031

**Published:** 2018-12-22

**Authors:** Jimin Jeon, Jiyoung Jang, Kyong Park

**Affiliations:** Department of Food and Nutrition, Yeungnam University, 280 Daehak-ro, Gyeognsan, Gyeongbuk 38541, Korea; jiminjeon@ynu.ac.kr (J.J.); today@ynu.ac.kr (J.J.)

**Keywords:** type 2 diabetes mellitus, calcium, yogurt, Korean adults

## Abstract

The effect of calcium consumption in the prevention of type 2 diabetes mellitus (T2DM) remains controversial, and depends on food calcium sources. This prospective study aimed to evaluate the association between calcium-rich food consumption and T2DM incidence among Korean adults. We analyzed the data of 8574 adults aged 40–69 years, without a history of T2DM, cardiovascular disease, and cancer at the baseline from the Korean Genome and Epidemiology Study. The consumption of calcium-rich foods was assessed using a validated semi-quantitative food frequency questionnaire. T2DM-related data were collected using biennial questionnaires, health examinations, and clinical tests. Hazard ratios (HRs) and 95% confidence intervals (CIs) were calculated using Cox proportional hazards regression models. In the multivariate-adjusted model, yogurt intake was inversely associated with T2DM risk (HR: 0.73; 95% CI: 0.61–0.88 in the fourth quartile as compared to the first quartile). However, the intakes of other calcium-rich foods, including milk and anchovies, were not significantly associated with T2DM risk. Yogurt may provide protective effects against T2DM in Korean adults, owing to the beneficial effects of probiotics. Further prospective large-scale cohort studies should be conducted to validate these findings.

## 1. Introduction

Type 2 diabetes mellitus (T2DM) is a chronic disease resulting from metabolic impairment owing to hyperglycemia. T2DM can be caused by either insulin resistance or impaired insulin secretion [1], and is considered a component of metabolic syndrome [2]. The global prevalence of T2DM has been increasing and is predicted to increase from 8.8% in 2015 to 10.4% by 2040 [3]. According to recent statistics from the Organisation for Economic Co-operation and Development (OECD), the incidence of T2DM in Korea is higher than the average in OECD countries [4]. The 2016 Korea National Health and Nutrition Examination Survey revealed that 11.3% of adults aged ≥30 years have T2DM [5]. T2DM causes several health complications, such as cardiovascular disease (CVD), an increase in morbidity and mortality, and a persistent aggravation of socioeconomic burden, making it a public health issue that demands constant attention worldwide [6].

To address this health issue, studies have proposed several dietary factors that may help prevent T2DM [7]. One of these factors is the intake of calcium, which is essential to insulin secretion and is involved in the mechanism of insulin action through the regulation of intracellular calcium levels [8]. According to a recent meta-analysis, calcium intake is associated with a lower risk of T2DM [9]. Similarly, epidemiological studies on the Korean population have reported an inverse association between dietary calcium intake and T2DM risk [10,11]. However, those previous analyses that used Korean data focused on single nutrient intake levels, without considering the type of food sources. In addition, they did not consider the screening of participants with implausible total energy intake values, or those with prevalent cardiovascular disease or cancer, which may affect interval validity in the analysis.

Calcium, as a nutrient, can be obtained through the consumption of various foods and dietary supplements, and its main source varies across countries. Unlike in Western countries, where dietary calcium is predominantly obtained from dairy products, in Korea, calcium sources include a variety of foods, including anchovies [12]. Previous reports have shown that the consumption of dairy products is lower among Koreans than in Western populations [13]. Non-dairy products such as kimchi, anchovies, tofu, radish leaves, soy, and sea mustard are the main sources of calcium among Koreans [12]. Furthermore, a calcium bioavailability analysis of calcium-rich foods showed that anchovies and tofu are as effective as milk [14].

Previous epidemiological studies that analyzed the effects of calcium-rich foods on T2DM showed inconsistent associations depending on the types of food sources, intake levels, ethnicity, and sex of the study populations [15,16,17,18]. For example, higher intakes of total dairy products were associated with decreased T2DM risk among Japanese women, but not among Japanese men [15]. The inverse association was evident among Australian men, but not Australian women [18]. The beneficial health effects of yogurt and fermented dairy products were also evident in Dutch [16] and Spanish [17] populations; however, higher intakes of full-fat dairy products were associated with T2DM prevalence [16].

While the association between calcium-rich food intake and T2DM risk has been reported primarily in the United States and Europe [19], few studies in Korean populations, in which the dietary calcium sources are more diverse than in Western countries, have focused on this association. Therefore, we aimed to prospectively examine the association between calcium-rich food consumption and T2DM incidence in Korean adults.

## 2. Materials and Methods

### 2.1. Study Population 

This study used data from the community-based Ansung-Ansan cohort, which was a part of the Korean Genome and Epidemiology Study. The survey methods used in this study have been reported in detail elsewhere [20]. In brief, the Ansung-Ansan cohort comprises 10,030 adult men and women aged 40–69 years residing in the Ansung and Ansan areas of the Gyeonggi Province. The baseline survey was conducted between 2001 and 2002 through the collection of data on the participants’ demographics, diet, lifestyle, environmental factors, and diseases. After the baseline survey, follow-up surveys were conducted every two years. The present study is based on the data obtained during the 2002–2012 follow-up. All surveys and data collection were conducted by trained researchers according to standardized protocols.

**Figure 1:**
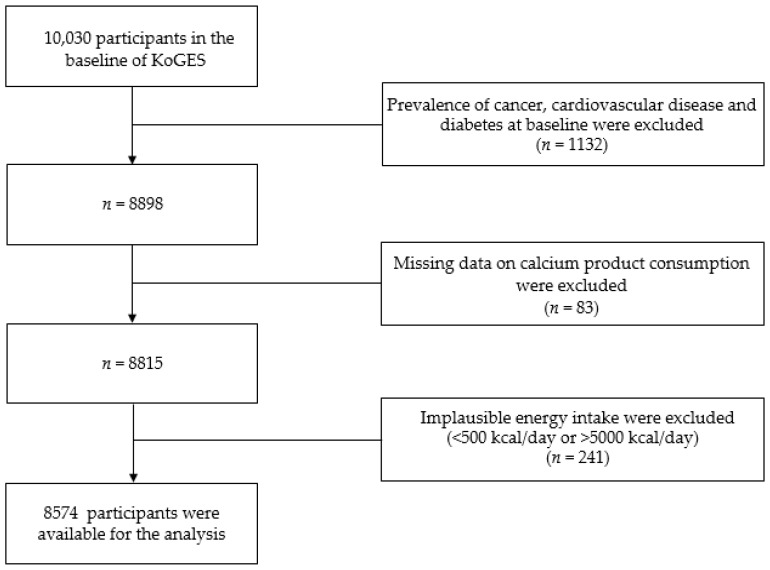
Flow chart showing the participant selection process. KoGES, Korean Genome and Epidemiology study.

Of the 10,030 people who participated in the Ansung-Ansan baseline survey, those with a previous diagnosis of T2DM, CVD, or cancer, and those taking medications or receiving treatments for these diseases (*n* = 1132) were excluded. We also excluded participants with missing data on the sources of calcium (*n* = 83) and those with a total daily energy intake <500 kcal or >5000 kcal (*n* = 241) [21]. Finally, 8574 participants were included in the current analysis (Figure 1).

Informed consent was obtained from all study participants. The methods used for data collection and analysis were approved by the Korea Centers for Disease Control and Prevention Institutional Review Board (IRB number: KU-IRB-15-EX-256-A-1) and the Yeungnam University Institutional Review Board (IRB number: 7002016-E-2016-003). 

### 2.2. General Participant Characteristics and Anthropometric Measurements

Data on the participants’ age, sex, residential area, education level, household income, physical activity, alcohol consumption, smoking status, and use of dietary supplements were collected using interviewer-administered questionnaires. Education level was classified as elementary school graduate or lower, middle school graduate, high school graduate, and college graduate or higher. Smoking status included current smoker, former smoker, and non-smoker. Based on alcohol consumption, participants were classified as drinkers and non-drinkers. Household income was categorized into the following four groups based on the average monthly income: <1 million Korean Republic Won (KRW), 1–<2 million KRW, 2–<4 million KRW, and ≥4 million KRW. Physical activity levels were quantified using metabolic equivalents of task (MET-hours/week), considering the weekly physical activity time and through the application of weights by exercise intensity [22]. Data on anthropometric parameters, such as weight and height, were collected by a trained technician, and the body mass index (BMI) was calculated by dividing the weight (kg) by the height squared (m^2^). The obesity status of the participants was reclassified using the BMI criteria of the World Health Organization for Asians [23], in which a BMI <25 kg/m^2^ indicates the absence of obesity and a value ≥25 kg/m^2^ indicates the presence of obesity.

### 2.3. Dietary Assessment

Dietary intake was assessed in the baseline survey (2001–2002) and the second follow-up survey (2005–2006) using a validated semi-quantitative food frequency questionnaire (SQFFQ). The validity and reproducibility of the SQFFQ have been reported previously [24]. In brief, questions on the frequency of food intake in the SQFFQ had nine possible responses: almost never, once per month, 2–3 times per month, 1–2 times per week, 3–4 times per week, 5–6 times per week, once per day, twice per day, and 3 times per day. Similarly, questions on the amount of food consumed had three possible responses: half a serving, one serving, and one-and-a-half servings. In this study, after the conversion of all frequencies to weekly frequencies, the amount of food consumed each time was used as a weight to determine the weekly intake (servings/week). Mean values were calculated across dietary data from the baseline survey and second follow-up survey to minimize misclassification. For missing dietary values during the second follow-up period, we imputed data using the fully conditional specification approach [25].

Based on Koreans’ calcium intake patterns and the Korean nutrient database, milk, yogurt, cheese, anchovies, lettuce, perilla leaves, sea mustard, laver, and beans were selected as the major calcium sources. The sum of the intake levels of each of these foods was used to calculate the total intake of calcium-rich foods. Of the dietary variables used as covariates, total vegetable intake was calculated from the sum of the intakes of 17 vegetables (Chinese cabbages, spinaches, lettuces, perilla leaves, bellflower roots, bean sprouts, bracken/sweet potato stems, red pepper leaves, leek/water dropworts/chamnamuls, cucumbers, carrots, onions, green peppers, pumpkins, courgettes, tomatoes, and mushrooms), excluding kimchi. Similarly, total fruit intake was calculated from the sum of the intakes of 11 fruits (strawberries, oriental melons/melons, watermelons, peaches, bananas, persimmons, mandarins, pears, apples, oranges, and grapes). Unprocessed beef and pork were defined as red meat, and ham and sausage were defined as processed meat.

### 2.4. Ascertainment of T2DM

T2DM-related data were collected through biennial questionnaires, health examinations, and clinical tests. All the procedures and clinical tests were performed by trained staff. T2DM cases were ascertained based on the following criteria: (1) fasting blood glucose (FBG) level ≥126 mg/dL or glycated hemoglobin (HbA1c) level ≥48 mmol/mol (6.5%) according to the criteria of the Korean Diabetes Association [26], (2) a doctor’s diagnosis, or (3) taking medication or undergoing current treatment for T2DM.

### 2.5. Statistical Analysis

The follow-up duration was calculated based on the time interval between the date of the baseline examination and (a) the date at which an event occurred for a participant with T2DM or (b) the last known date of life in those without T2DM. A chi-square test for categorical variables and a linear regression analysis for continuous variables were performed to compute the mean and standard error for each quartile of calcium-rich food intake. Energy-adjusted food and nutrient intakes were calculated using the residual method [21].

Cox proportional hazards regression analysis was performed to calculate hazard ratios (HRs) and their 95% confidence intervals (CIs). Multiple potential confounding variables and effect modifiers were determined based on preliminary analyses and previous studies [16,27,28]. The *p* for trend was calculated using the median of the food intake quartiles as a continuous variable. Interactions with demographic and lifestyle factors were tested using linear and Cox proportional hazards regression. We then built three covariate models: (a) model 1 that was unadjusted; (b) model 2 that was adjusted for age, sex, BMI, residential area, education level, household income, physical activity, alcohol consumption, and smoking status; and (c) model 3 that was additionally adjusted for a history of hypertension, family history of T2DM, use of antihypertensive medication, use of dietary supplements, and intakes of vegetables, fruits, red meat, processed meat, soft drinks, coffee, and tea. Statistical Analysis System version 9.4 (SAS Institute Inc., Cary, NC, USA) was used for all statistical analyses, with significance defined as *p* < 0.05.

## 3. Results

During the average follow-up of 7.3 years, 1173 incident cases of T2DM were identified. The mean age of the participants at the baseline was 51.7 ± 0.1 years. Table 1 shows the general characteristics of the participants based on the quartiles of total calcium-rich food intake. A higher intake of calcium-rich foods was observed among women (*p* < 0.001), residents of Ansan (*p* < 0.001), individuals with moderate physical activity (*p* < 0.001), non-drinkers (*p* < 0.001), non-smokers (*p* < 0.001), and users of dietary supplements (*p* < 0.001). A higher intake of calcium-rich foods was also significantly associated with lower education levels (*p* < 0.001) and lower household income (*p* < 0.001). 

The HRs of incident T2DM based on the quartiles of calcium-rich food intake are shown in Table 2. In the minimally and fully adjusted models, a higher intake of yogurt was associated with a decreased risk of incident T2DM (model 1 HR: 0.63; 95% CI: 0.53–0.74 and model 3 HR: 0.73; 95% CI: 0.61–0.88; *p* for trend: 0.01). Meanwhile, the intakes of milk, anchovies, and other sources (cheese, beans, perilla leaves, sea mustard, laver, and lettuce) of calcium were not significantly associated with T2DM risk.

**Figure 2:**
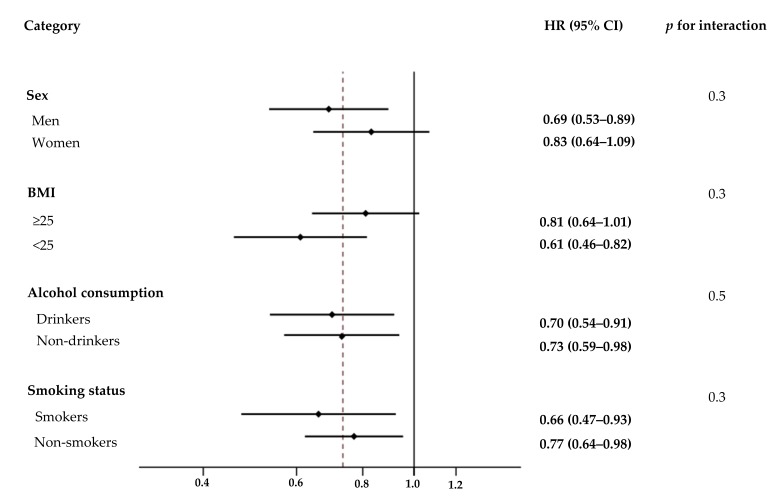
Effect of various demographic factors on the association between yogurt intake and risk of type 2 diabetes mellitus (T2DM). Hazard ratios (HRs) and 95% confidence intervals (CIs, shown in parentheses) of T2DM in the fourth quartile of energy-adjusted yogurt intake were compared to the first quartile based on sex, body mass index, alcohol consumption, and smoking status. Values were adjusted for the listed variables simultaneously and other potential confounders which included age, residential area, education level, household income, physical activity, history of hypertension, family history of type 2 diabetes, use of antihypertensive medication, use of dietary supplements, and intakes of vegetables, fruits, red meat, processed meat, soft drinks, coffee, and tea. BMI, body mass index.

The association between yogurt intake and T2DM incidence was further analyzed by stratifying multiple demographic factors such as sex, BMI, alcohol consumption, and smoking status (Figure 2). The association between yogurt intake and T2DM risk was not affected by sex (*p* for interaction = 0.3), BMI (*p* for interaction = 0.3), alcohol consumption (*p* for interaction = 0.5), or smoking status (*p* for interaction = 0.3). 

## 4. Discussion

This study prospectively examined the association between calcium-rich food intake and T2DM among Korean adults using community-based Ansung-Ansan cohort data. Of the calcium-rich food intakes evaluated, although higher yogurt intakes were associated with a decreased T2DM risk, none of the other foods showed any association. Yogurt, as a fermented dairy product, is beneficial to health. Besides being a source of protein, minerals, and vitamins, many types of yogurt contain high concentrations of probiotics such as *Lactobacillus delbrueckii* subsp. *bulgaricus* and *Streptococcus thermophilus* [29], which are live microorganisms that, when administered in adequate amounts, positively affect health [30]. At appropriate doses, probiotics can alleviate intestinal conditions such as inflammatory bowel disease [31], and the lactic acid produced during yogurt fermentation increases protein and calcium absorption, thereby promoting bone mineralization [32]. Moreover, probiotics have anti-inflammatory [31] and antioxidant effects [33] associated with T2DM prevention. An excessive accumulation of reactive oxygen species results in oxidative stress, inflammation that interferes with insulin pathways, and increased insulin resistance [34]. Probiotics reduce oxidative stress and the degree of inflammation, in turn reducing the rate of insulin resistance. Randomized controlled trials (RCTs), including T2DM participants, have reported significantly lower HbA1c and FBG levels, as well as insulin resistance rates in participant groups that consume probiotics [35,36]. Participants who consumed yogurt had metabolic profiles with significantly lower FBG levels and reduced insulin resistance rates than those who did not consume the same [37]. Moreover, the vitamin D and magnesium found in yogurt also have beneficial effects in T2DM prevention [38,39].

Previous studies that analyzed the association between yogurt intake and T2DM risk showed consistent results [16,17,40]. In a study of American women, higher yogurt intake levels were associated with a lower T2DM risk (HR: 0.82; 95% CI: 0.70–0.97) [40]. This association was evident regardless of whether the yogurt was low-fat (HR: 0.68; 95% CI: 0.47–0.97) or regular (HR: 0.66; 95% CI: 0.47–0.92) [17]. Furthermore, a recent pooled analysis confirmed that yogurt intake reduces T2DM risk [19]. However, besides yogurt, no other dairy products had any effect on T2DM prevention. In a meta-analysis, neither milk (standard, high-fat, and low-fat) nor cheese was significantly associated with T2DM risk [19]. Although no mechanism clearly explains the differences in the preventive effects of different dairy products, the beneficial effects of yogurt may be attributed to the presence of probiotics. Further research is needed to investigate the mechanisms underlying these differences.

This study has several limitations. Although we adjusted for multiple confounding factors that could affect T2DM risk, the results could have been affected by other residual confounding factors that we could not measure or were unaware of. Additionally, we could not perform a more detailed analysis based on contents such as fat and probiotics, as dairy products are sold without distinction of fat content in the Korean market, and even if the contents and concentrations of probiotics are different across various brands and products, the relevant data are insufficient [41]. Lastly, as the study participants were limited to residents of the Ansung and Ansan area in South Korea, it may be difficult to generalize the study’s results for other populations. Despite these limitations, we estimated the dietary intakes using repeated measurements to minimize measurement errors, and prospectively analyzed the association between calcium-rich food intake and T2DM risk in the Korean population. 

## 5. Conclusions

In conclusion, this study’s results show that among the intakes of several different calcium-rich foods, those of yogurt alone reduce T2DM risk. Further large-scale RCTs should be conducted, considering the composition of yogurt (sugar, fat, and probiotics). 

## Figures and Tables

**Table 1:** Baseline characteristics of participants according to quartiles (Q) of energy-adjusted total calcium product intake.

	Total Calcium Product Intake (Quartile)				*p* value ^a^
	Q1	Q2	Q3	Q4	
***n***	2143	2144	2144	2143	
**Total calcium product intake (servings/week)**	7.0	12.2	17.6	26.9	
**Male**	1272 (59.4)	1190 (55.5)	1009 (47.1)	613 (28.6)	<0.001
**Age (years)**	51.4 ± 0.2	50.7 ± 0.2	51.4 ± 0.2	53.3 ± 0.2	<0.001
**Residential area**					<0.001
Ansung	1318 (61.5)	902 (42.1)	867 (40.4)	1033 (48.2)	
Ansan	825 (38.5)	1242 (57.9)	1277 (59.6)	1110 (51.8)	
**Education level**					<0.001
Elementary school graduation or lower	744 (35.0)	609 (28.6)	635 (29.8)	770 (36.1)	
Middle school graduation	494 (23.2)	470 (22.1)	474 (22.2)	522 (24.5)	
High school graduation	629 (29.6)	725 (34.0)	699 (32.8)	594 (27.8)	
College graduation or higher	260 (12.2)	328 (15.4)	325 (15.2)	248 (11.6)	
**Monthly household income (KRW)**					<0.001
<1,000,000	861 (40.7)	643 (30.3)	625 (29.5)	753 (35.7)	
1,000,000–<2,000,000	626 (29.6)	640 (30.2)	591 (27.9)	639 (30.3)	
2,000,000–<4,000,000	525 (24.8)	660 (31.2)	687 (32.5)	563 (26.7)	
≥4,000,000	103 (4.9)	176 (8.3)	213 (10.1)	153 (7.3)	
**Body mass index (kg/m^2^)**	24.5 ± 0.1	24.5 ± 0.1	24.6 ± 0.1	24.5 ± 0.1	0.3
**Physical activity ^b^**					<0.001
Low	674 (31.6)	773 (36.3)	713 (33.7)	678 (31.9)	
Mid	563 (26.4)	719 (33.8)	760 (35.9)	789 (37.1)	
High	894 (42.0)	638 (30.0)	646 (30.5)	661 (31.1)	
**Alcohol consumption (yes)**	1113 (52.2)	1152 (53.9)	1050 (49.2)	808 (37.9)	<0.001
**Smoking status**					<0.001
Non-smokers	1031 (48.5)	1151 (53.9)	1260 (59.5)	1557 (73.5)	
Former smokers	361 (17.0)	378 (17.7)	331 (15.6)	217 (10.3)	
Smokers	736 (34.6)	606 (28.4)	528 (24.9)	344 (16.2)	
**Dietary supplement use (yes)**	277 (13.1)	347 (16.4)	409 (19.4)	541 (25.3)	<0.001

Values are mean ± standard error or *n* (%); KRW is Korean Republic Won; ^a^ *p* values are derived from χ^2^ test for categorical variables and from generalized linear regression analysis for continuous variables; ^b^ Physical activity was categorized into three groups, according to tertile of metabolic equivalents (MET)-hours/week.

**Table 2:** Hazard ratio (95% confidence interval) for diabetes risk according to energy-adjusted calcium product intake level.

	Frequency of Consumption (Quartile)				*p* for Trend
	Q1	Q2	Q3	Q4	
**Milk**					
Median, servings/week	0	0.3	2.0	8.0	
Case/*n*	307/2139	305/2140	271/2140	288/2139	
Person year	13,815	15,407	15,750	15,312	
Model 1	1	0.89 (0.76, 1.05)	0.78 (0.66, 0.91)	0.85 (0.72, 1.00)	0.2
Model 2	1	1.00 (0.85, 1.18)	0.95 (0.80, 1.12)	1.04 (0.88, 1.23)	0.6
Model 3	1	1.05 (0.88, 1.25)	0.95 (0.79, 1.13)	1.05 (0.88, 1.25)	0.7
**Yogurt**					
Median, servings/week	0	0.3	1.3	5.0	
Case/*n*	323/2139	311/2139	295/2040	241/2139	
Person year	13,246	15,558	15,751	15,708	
Model 1	1	0.82 (0.70, 0.96)	0.77 (0.66, 0.90)	0.63 (0.53, 0.74)	<0.001
Model 2	1	0.83 (0.71, 0.97)	0.84 (0.72, 0.99)	0.72 (0.60, 0.86)	0.002
Model 3	1	0.82 (0.70, 0.97)	0.84 (0.70, 0.99)	0.73 (0.61, 0.88)	0.01
**Anchovies**					
Median, servings/week	0.2	0.8	1.8	4.6	
Case/*n*	310/2141	291/2141	282/2142	288/2141	
Person year	13,596	15,607	15,830	15,281	
Model 1	1	0.82 (0.70, 0.96)	0.78 (0.66, 0.92)	0.83 (0.70, 0.97)	0.1
Model 2	1	0.93 (0.79, 1.10)	0.86 (0.73, 1.02)	0.89 (0.75, 1.05)	0.3
Model 3	1	0.91 (0.77, 1.08)	0.82 (0.69, 0.98)	0.87 (0.73, 1.04)	0.2
Other calcium sources *					
Median, servings/week	4.0	7.1	10.5	17.6	
Case/*n*	327/2143	296/2144	257/2144	293/2143	
Person year	14,635	15,564	15,499	14,688	
Model 1	1	0.83 (0.70, 0.97)	0.81 (0.69, 0.95)	1.07 (0.91, 1.25)	0.1
Model 2	1	0.91 (0.77, 1.08)	0.86 (0.72, 1.02)	1.05 (0.89, 1.24)	0.4
Model 3	1	0.86 (0.72, 1.03)	0.78 (0.85, 0.93)	0.97 (0.81, 1.16)	1.0
**Total calcium products**					
Median, servings/week	7.0	12.2	17.6	26.9	
Case/*n*	341/2143	276/2144	284/2144	272/2143	
Person year	14,526	15,512	15,395	14,953	
Model 1	1	0.77 (0.66, 0.91)	0.82 (0.70, 0.97)	0.93 (0.79, 1.08)	0.8
Model 2	1	0.85 (0.72, 1.00)	0.88 (0.74, 1.04)	0.98 (0.83, 1.16)	0.9
Model 3	1	0.82 (0.69, 0.97)	0.83 (0.70, 0.99)	0.92 (0.77, 1.11)	0.7

* Sum of cheese, tofu, perilla leaves, sea mustard, laver, and lettuce; Model 1: unadjusted; Model 2: adjusted for age, sex, body mass index, residential area, education level, household income, physical activity, alcohol consumption, and smoking status; Model 3: additionally adjusted for history of hypertension, family history of type 2 diabetes, use of antihypertensive medication, use of dietary supplements, and intakes of vegetables, fruits, red meat, processed meat, soft drinks, coffee, and tea; Q, quartile.
